# A Versatile SERS Sensor for Multiple Determinations of Polycyclic Aromatic Hydrocarbons and Its Application Potential in Analysis of Fried Foods

**DOI:** 10.1155/2020/4248029

**Published:** 2020-08-03

**Authors:** Shi Wang, Jie Cheng, Caiqin Han, Jianchun Xie

**Affiliations:** ^1^Institute of Quality Standards and Testing Technologies for Agro-products, Chinese Academy of Agricultural Sciences, Beijing 100081, China; ^2^Jiangsu Key Laboratory of Advanced Laser Materials and Devices, School of Physics and Electronic Engineering, Jiangsu Normal University, Xuzhou 221116, China; ^3^Beijing Advanced Innovation Center for Food Nutrition and Human Health, Beijing Technology & Business University (BTBU), Beijing 100048, China

## Abstract

Polycyclic aromatic hydrocarbons (PAHs), due to their high hydrophobicity, have low affinity for metallic SERS-active surfaces, which leads to their low SERS detection sensitivity. Various functional groups have been used to improve the affinity of metallic substrates towards the target PAHs. However, a large portion of the signals generated from the “first-layer effect” of the functionalized substrates may complicate the spectrum, leading to a distortion in the assignment of the intrinsic SERS fingerprints of PAHs. In this study, a SERS sensor composed of Au nanoparticles (AuNPs) and reoxidized graphene oxide (rGO) was developed for the simultaneous determination of 16 EPA priority PAHs. The synthesis of the rGO/AuNP substrate can be realized without a complicated modification process. All the 16 PAHs could be identified based on their characteristic peaks in the presence of the composited substrate, with estimated LOD as low as 0.2–2 ng·mL^−1^. The binary linear regression was optimized as the fitting model for all PAHs except for benzo(k)fluoranthene, with the linear correlation coefficient ranging from 0.9889 to 0.9997. Based on the developed SERS substrates and sample pretreatment, the characteristic SERS peaks of four PAHs in Chinese traditional fried food (youtiao) were identified without any background interference. The whole detection process only takes approximately 15 minutes. The results demonstrate the potential of the multicomponent on-field detection of PAHs.

## 1. Introduction

Polycyclic aromatic hydrocarbons (PAHs) consisting of fused aromatic rings without any substitution groups are well-known carcinogens and classified as a group of widespread persistent organic pollutants. PAHs are ubiquitously present in the atmosphere, water, and soil and generated during incomplete combustion of organic matters [1]. PAHs pose risk to human health due to their accumulation, migration, and transformation in the food chain. In addition, the processing and cooking of food at high temperatures can also contribute to the occurrence of PAHs. For instance, deep-frying in edible oils leads to high risk of exposure to PAHs [2, 3]. Based on the risk assessment by the United States Environmental Protection Agency, in 1976, 16 PAHs (Table 1) were selected as “priority polycyclic aromatic compounds” on the basis of their toxicity and the risks to human health [4, 5].

The traditional analytical methods for PAHs usually include gas chromatography-mass spectrometry (GC-MS) [6], high-performance liquid chromatography (HPLC) [7], gas chromatography-triple quadrupole mass spectrometry (GC-MS/MS) [8, 9], and comprehensive two-dimensional gas chromatography with time-of-flight mass spectrometry (GC × GC-TOF MS) [10]. The laborious pretreatment steps and the large-scale instruments often required for these methods are only suitable for laboratory analysis. In recent years, some novel screening methods such as fluorescence microscopy [11], ion mobility spectrometry [12], real-time immuno-PCR [13], electrochemical sensor [14–17], pH change sensor [18], and surface-enhanced Raman spectroscopy (SERS) [19, 20] have emerged.

Surface-enhanced Raman scattering (SERS), as a powerful analytical technique, can provide the fingerprint of a molecule at the trace level concentrations. Under the synergism of electromagnetic and chemical effects, the Raman signal of the target compound can be greatly enhanced. Due to their high hydrophobicity, PAHs have a low affinity for metallic SERS-active surfaces, which results in their low SERS detection sensitivity. Surface functionalization of the substrates has been extensively investigated to improve their affinity towards the target PAH molecules. Various functional groups or ligands such as thiols [21], alkyl chains [22], calixarenes [23], cyclodextrin derivatives [24], dopamine [25], inositol hexaphosphate [26], and antibodies [27] have been investigated for surface modification. However, a large portion of the signals generated from “first-layer effect” of the functionalized substrates may complicate the spectrum leading to the distortion and wrong assignment of the intrinsic SERS fingerprints of target PAHs [28]. In addition, lower limit of detections (LODs) can also be realized on substrates with specific geometries such as core-shell MOF/Ag nanoparticle composites [29], Au nanoparticles grafted on [18] or doped with [30] Fe_3_O_4_, and gold nanoparticles embedded in alginate gel network [31]. These composites offered enhanced metal-analytes interactions by adsorbing target molecules in close proximity to the “hot spots” at the junctions between nanoparticles. Despite the ultrahigh enhancement effects obtained from these hybrid SERS substrates, their synthesis was generally complicated. The substrates were often required to be immersed in PAH solutions for a certain period (10–60 min) to allow the interaction between the target PAHs and the substrates. Most of the substrates reported earlier were prepared to detect only one or more minority PAHs. Very few studies have focused on the detection of several EPA priority PAHs.

In this study, a versatile SERS sensor composed of Au nanoparticles (AuNPs) and reoxidized graphene oxide (rGO) was developed for the simultaneous detection of 16 EPA priority PAHs. The use of GO is favorable for the adsorption of PAHs because of the novel chemical and physical properties of graphene. The PAHs could be differentiated based on their characteristic Raman shifts. Through sample pretreatment and SERS enhancement of the stable and reproducible SERS sensor, the characteristic SERS peaks of four PAHs in a sample of youtiao, a typical Chinese fried food, were identified without any background interference. The whole detection process only takes about 15 min. The study offered a new method to tailor the structure of graphene-based SERS substrates for on-site screening or point-of-care applications for the detection of PAHs.

## 2. Materials and Methods

### 2.1. Materials and Reagent

Chloroauric acid tetrahydrate (HAuCl_4_·4H_2_O, >47.8%), sodium citrate, and hydrogen peroxide (30%) were of analytical grade and purchased from Sinopharm Chemical Reagent Co., Ltd (Beijing, China). Graphene oxide solution (GO, 1 mg mL^−1^) was obtained from XF Nano Co., Ltd (Shanghai, China). Naphthalene (1000 ng *μ*L^−1^, methanol) was acquired from J & K Scientific Co., Ltd (Beijing, China). Acenaphthene (100 ng *μ*L^−1^, acetonitrile), pyrene (100 ng *μ*L^−1^, acetonitrile), anthracene (10 ng *μ*L^−1^, acetonitrile), acenaphthylene (100 ng *μ*L^−1^, acetonitrile), chrysene (100 ng *μ*L^−1^, acetonitrile), fluorene (100 ng *μ*L^−1^, acetonitrile), phenanthrene (100 ng *μ*L^−1^, acetonitrile), benzo(a)pyrene (100 ng *μ*L^−1^, methanol), fluoranthene (100 ng *μ*L^−1^, methanol), benzo(a)anthracene (100 ng *μ*L^−1^, acetonitrile), dibenz(a, h)anthracene (100 ng *μ*L^−1^, acetonitrile), benzo(b)fluoranthene (100 ng *μ*L^−1^, acetonitrile), benzo(k)fluoranthene (10 ng *μ*L^−1^, acetonitrile), benzo(g, h, i)perylene (10 ng *μ*L^−1^, acetonitrile), and indeno(1, 2, 3-cd)pyrene (100 ng *μ*L^−1^, cyclohexane) were purchased from ANPEL Laboratory Technologies Inc. (Shanghai, China).

### 2.2. Preparation and Characterization of SERS Sensor

The SERS sensor was prepared by a one-pot synthesis as described previously with some modifications [32]. Briefly, 4 mL of 1 mg·mL^−1^ GO solution and 4 mL of 30% H_2_O_2_ solution were mixed into 36 mL of distilled water (DI) at room temperature. After stirring overnight, 4 mL of the mixture was transferred into 80 mL of DI water. With constant stirring at 800 rpm, 0.5 mL of 1 wt. % HAuCl_4_ and 0.5 mL of 30% H_2_O_2_ solution were added into the above mixture sequentially. Following the addition of 0.3 mL of 1 wt. % aqueous sodium citrate, the resulting solution was heated to boiling and reacted for 10 min. After removing the heating source, the final rGO/AuNP particles were continuously stirred and cooled down to room temperature. The products were collected by centrifugation at 2800 rpm for 5 min, washed, and dispersed in DI water. The prepared rGO/AuNP solution was stored at 4°C and gently vortexed for 1 min before every use. The morphology of the substrate was characterized by transmission electron microscopy (TEM) (Hitachi).

### 2.3. Detection of 16 PAHs

The calibration solutions of all 16 PAHs were diluted with methanol. Approximately 600 *µ*L of the rGO/AuNP colloid was gently mixed with a 60 *µ*L solution containing various concentrations of PAHs, for 10 s. Then, the mixture was exposed to a 785 nm incident laser for 10 s at 200 mW power assembled on the portable Raman spectrometer (Raman Tracer-200-HS). The Raman spectrum obtained in the 500–2500 cm^−1^ range was an average of three scans and processed with Raman Analyzer software from Leap-SCI Technologies, Inc. The Savitzky–Golay second derivative transformation was used to remove the background signal, and other preprocessing algorithms such as smoothening and polynomial subtraction were also used [33].

### 2.4. Sample Detection

A ceramic homogenizer for QuECHERS and 5 mL of hexane were mixed with 1.0 g of the ground sample. After vigorous shaking for 1 min, 10 mL of a mixture of water and acetonitrile (v/v = 1 : 1) was added. Subsequently, MgSO_4_ (4.2 g) and NaCl (0.6 g) were added and the mixture was shaken for 1 min and centrifuged at 12000 rpm for 30 s to separate the PAHs into the acetonitrile phase. 1 mL of the subnatant was added into a prepared mix of the QuECHERS pouch (50 mg PSA + 150 mg MgSO_4_) and hand-shaken for 30 s. Centrifugation for 60 s at 10000 rpm resulted in the formation of two separate layers. Further, 60 *µ*L of the extracted supernatant was then vigorously mixed with 600 *µ*L of the rGO/AuNP colloid. The mixture was detected with a portable Raman instrument under similar conditions as described in Section 2.3.

## 3. Results and Discussion

### 3.1. The Morphology of the Substrate and Its Sensing Performance

**Figure 1:**
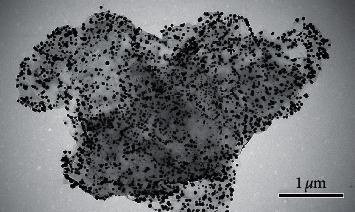
TEM image of the prepared rGO/AuNPs substrate.

**Figure 2:**
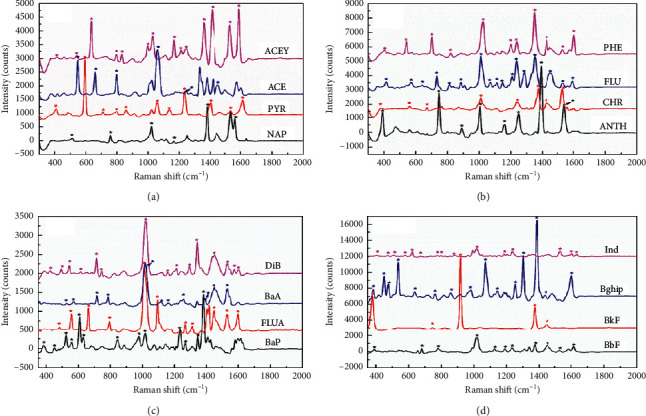
SERS spectra of 16 PAHs (*C* = 100 ng mL^−1^) in the presence of rGO/AuNP substrates.

The oxygen functionalities may provide reactive sites for the nucleation and growth of AuNPs. The electrostatic interactions between the oxygen groups (carboxylic and hydroxyl) on the surface of rGO, resulting from the reoxidation operation, made the AuNPs anchor more closely on the surface of the GO sheets (Figure 1). With the synergism obtained between the aggregated AuNPs and the graphene-enhanced Raman scattering (GERS) effect of graphene, the composited substrate demonstrated good SERS sensing performance for all 16 PAHs (Figure 2). Simultaneously, AuNPs with the same diameters were prepared based on the synthesis conditions described in Section 2.2. Compared to the AuNPs, the substrates consisting of AuNPs supported on twice-oxidized GO (rGO/AuNP) showed an excellent enhancement of the Raman scattering when tested with the 16 PAHs (Figure S1). There are fewer number of Raman peaks in the presence of AuNPs, compared with the apparently characteristic Raman shift obtained from rGO/AuNP. It is supposed that at the initial preparation step, the twice oxidization treatment for GO favors the increase of density of oxygen functional groups (carboxylic and hydroxyl) on the surface. The oxygen groups are responsible for the attachment of the free Au (III) ions in the solution because of electrostatic interactions followed by nucleation. A higher density of AuNPs deposited on the surface of GO leads to much narrower interparticle distance and creates more “hot spots.” LSPR from hot spots brings about greater enhancement of the PAH signal.

The characteristic SERS peaks were identified by the asterisk mark, and the corresponding vibrational modes were assigned as shown in Tables S1–S16. The prepared composited SERS substrates demonstrated high SERS activity towards all 16 PAHs, especially in the regions of 1200–1600 cm^−1^, which was mainly attributed to C-H bending modes. Compared with the C-C bending appearing in the regions of 300–1000 cm^−1^, C-H bending modes showed higher peak intensity, primarily due to their higher polarizability [34]. Meanwhile, PAHs with a larger number of C-H groups, such as DiB, BbF, BkF, BaP, and Bghip, have a richer number of Raman fingerprints compared with PAHs with fewer C-H groups (NAP). Furthermore, PAHs with a more symmetric molecular structure like BkF and CHR showed much more resolved peaks than other PAHs with asymmetric structures. In previous studies, the SERS substrates were often immersed in the target PAH solutions for a certain period of time (e.g., 30 min or 50 min) to allow the PAHs to partition to the substrate surface [24, 25]. Another common method was the solvent evaporation after dropping of PAHs onto the substrate surface, which usually required more than 10 min [22]. However, the sensing performance in the present study was realized as soon as the interaction between the PAHs solutions and the rGO/AuNP substrate was over, without the requirement any equilibration period, resulting in improvement of the overall detection rapidity.

### 3.2. Calibration Curve and Limits of Detection (LOD)

To establish the quantitative calculation model for PAHs, different methods including linear regression and binary linear regression were used. Three PAHs including NAP, PYR, and ACE were analyzed. Meanwhile, the quantitative calculation model of the other 13 PAHs is listed in Table S17.

**Figure 3:**
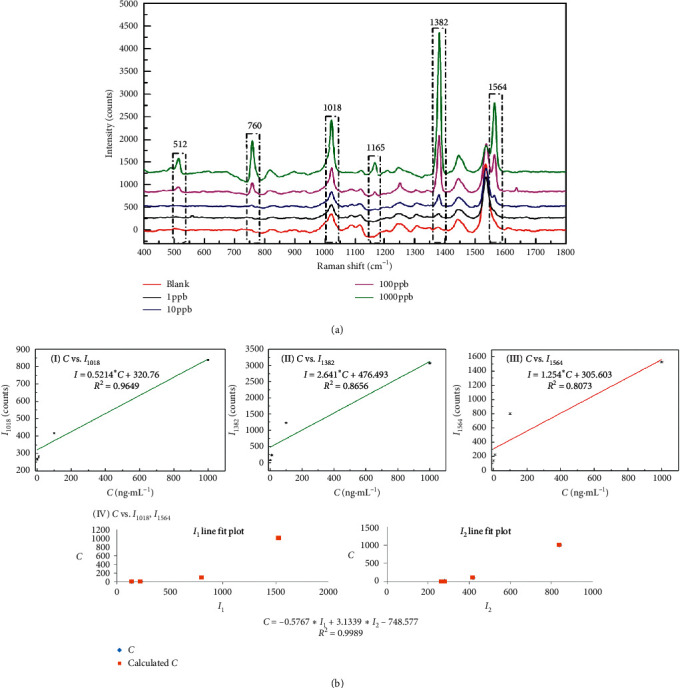
(a) The SERS spectra of NAP with different concentrations in the presence of rGO/AuNP; (b) fitting results of peak intensity (*I*) and concentration (*C*) based on different characteristic peaks. (I) Linear regression with the characteristic peak at Δ*v* = 1018 cm^−1^; (II) linear regression with the characteristic peak at Δ*v* = 1382 cm^−1^; (III) linear regression with the characteristic peak at Δ*v* = 1564 cm^−1^; (IV) binary linear regression with the characteristic peak at Δ*v* = 1018 cm^−1^ and Δ*v* = 1564 cm^−1^.

NAP: as shown in Figure 3, the characteristic SERS peaks of NAP appeared at Δ*v* = 512, 760, 1018, 1165, 1382, and 1564 cm^−1^. Their intensities of the characteristic peak (*I*_512_, *I*_760_, *I*_1018_, *I*_1165_, *I*_1382_, and *I*_1564_) gradually increased with increasing concentration of NAP from 1 to 1000 ng mL^−1^. However, *I*_1018_, *I*_1382_, and *I*_1564_ have displayed a more regular variation trend and were selected for quantitative analysis. First, the correlation between *I* and *C* was established using linear regression. Figures 3(a)–3(c) show the linear correlations between *C* and *I*_1018_, *I*_1382_, and *I*_1564_, and the linear correlation coefficient (*R*^2^) was 0.9646, 0.8656, and 0.8073, respectively. Second, two characteristic peaks at Δ*v* = 1018 and 1564 cm^−1^ were selected and the correlation between *I*_1018_, *I*_1564_, and *C* was established by the binary linear regression (Figure 3(b) IV). The correlation coefficient (*R*^2^) was 0.9989. The results of fitting results showed that the model using the binary linear regression based on *I*_1018_ and *I*_1564_ is the most suitable for the quantitative detection of NAP.

**Figure 4:**
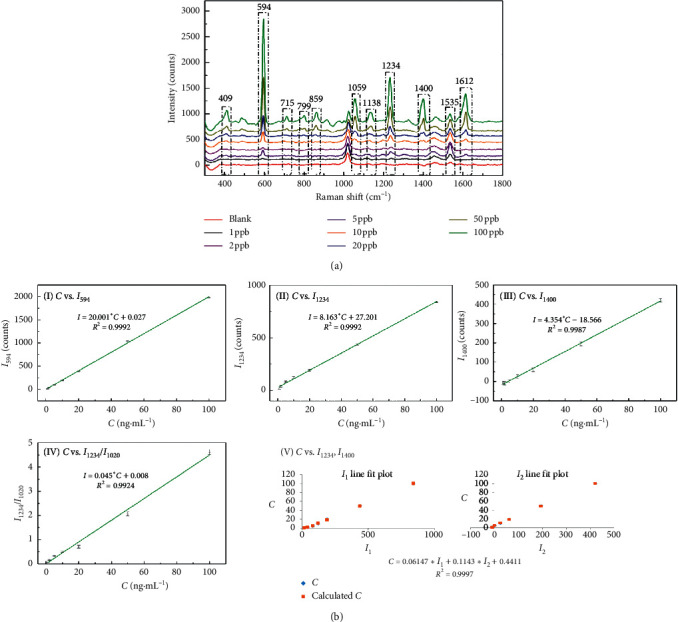
(a) The SERS spectra of PYR with different concentrations in the presence of rGO/AuNP; (b) fitting results of peak intensity (*I*) and concentration (*C*) based on different characteristic peaks. (I) Linear regression with the characteristic peak at Δ*v* = 594 cm^−1^; (II) linear regression with the characteristic peak at Δ*v* = 1234 cm^−1^; (III) linear regression with the characteristic peak at Δ*v* = 1400 cm^−1^; (IV) linear regression with the intensity ratio between Δ*v* = 1234 cm^−1^ and Δ*v* = 1020 cm^−1^; (V) binary linear regression with the characteristic peak at Δ*v* = 1234 cm^−1^ and Δ*v* = 1400 cm^−1^.

PYR: three characteristic peaks at Δ*v* = 594, 1234, and 1400 cm^−1^ were selected as the quantitative peaks. The linear correlations between *I* and *C* are shown in Figure 4. The binary linear regression between *I*_1234_, *I*_1400_, and *C* is also shown in Figure 4(b) V. Interestingly, the peak at Δ*v* = 1020 cm^−1^ was exploited as an internal reference and the calculated ratios of peaks intensities (*I*_1234 cm_^−1^/*I*_1020 cm_^−1^) showed good linear correlation vs. *C* (*R*^2^ = 0.9924). Similar to NAP, two characteristic peaks at Δ*v* = 1234 and 1400 cm^−1^ were selected and the correlation between *I*_1234_, *I*_1400_, and C was established by the binary linear regression (Figure 4(b) V). The correlation coefficient (*R*^2^) was 0.9997. The results of fitting results showed that the model using the binary linear regression based on *I*_1234_ and *I*_1400_ is the most suitable for the quantitative detection of PYR.

**Figure 5:**
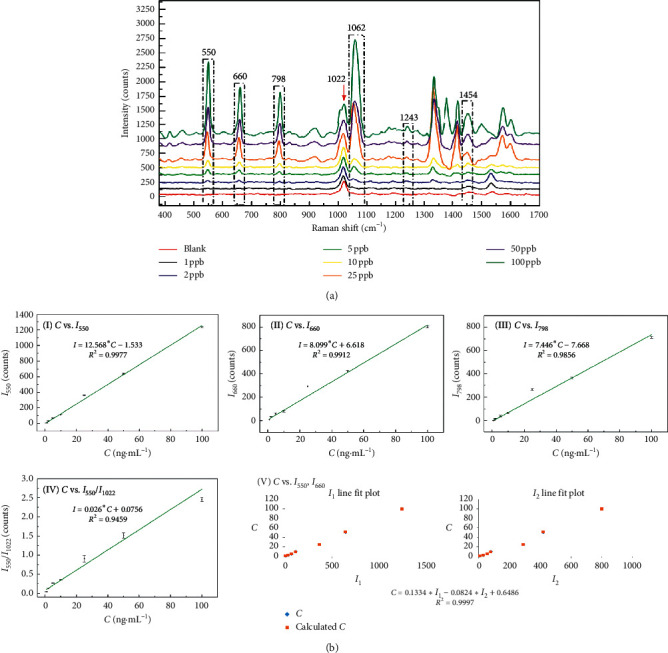
(a) The SERS spectra of ACE with different concentrations in the presence of rGO/AuNP; (b) fitting results of peak intensity (*I*) and concentration (*C*) based on different characteristic peaks. (I) Linear regression with the characteristic peak at Δ*v* = 550 cm^−1^; (II) linear regression with the characteristic peak at Δ*v* = 660 cm^−1^; (III) linear regression with the characteristic peak at Δ*v* = 798 cm^−1^; (IV) linear regression with the intensity ratio between Δ*v* = 550 cm^−1^ and Δ*v* = 1022 cm^−1^; (V) binary linear regression with the characteristic peak at Δ*v* = 550 cm^−1^ and Δ*v* = 660 cm^−1^.

ACE: the characteristic peaks at Δ*v* = 550, 660, and 798 cm^−1^ were obvious in ACE standard solutions at various concentrations. The linear correlations between *C* and *I*_550_, *I*_660_, and *I*_798_ are shown in Figure 5. The *R*^2^ values were 0.9977, 0.9912, and 0.9856, respectively. Similar to PYR, the peak at Δ*v* = 1022 cm^−1^ was exploited as an internal reference, and the linear correlation between the ratio of peaks intensities (*I*_550 cm_^−1^/*I*_1022 cm_^−1^) and *C* was calculated as *R*^2^ = 0.9459. The binary linear regression between *I*_550_, *I*_660_, and *C* was also established in Figure 5(b) V. The results showed that the model using the binary linear regression based on *I*_550_ and *I*_660_ is the most suitable for the quantitative detection of ACE.

In previous studies, the main principle behind quantitative peak selection was based on the most intense Raman band. However, in this study, the concentration-dependent response of PAHs was described based on different modes, especially the binary linear regression. Table S17 shows that the binary linear regression model was the most optimized fitting model for all PAHs except for BkF, while the linear correlation coefficient ranged from 0.9889 to 0.9997. In addition, compared with the previously reported studies, the quantitative calculation range in the present study was larger (10–100 *μ*M level). As we know, because of the saturation of “hot spots,” the maximum concentration adsorbed on the substrates will reach a plateau. The higher density of hot spots may lead to more influenced targeting of molecules. It was indicated that the loading of AuNPs from the capture of rGO improves the detected mass sensitivity of target PAHs, which was attributed to the combination of electromagnetic effects of AuNPs and chemical interactions between the rGO surface and PAHs [35].

The LOD was measured based on the 3*σ* methods [36], in which the intensities of characteristic SERS peaks were compared with a threshold value determined by three times the standard deviation of the spectral intensity fluctuation at a featureless spectra region (1700–1800 cm^−1^). The LODs of 16 PAHs are summarized in Table S17. Although the designed substrates have demonstrated SERS activity towards all 16 PAHs, the LODs (0.2–2 ng·mL^−1^) were relatively higher than that reported in the literature [19]. Future research will be directed towards the improvement of the sensitivity of the composited substrate.

### 3.3. Stability and Reproducibility

**Figure 6:**
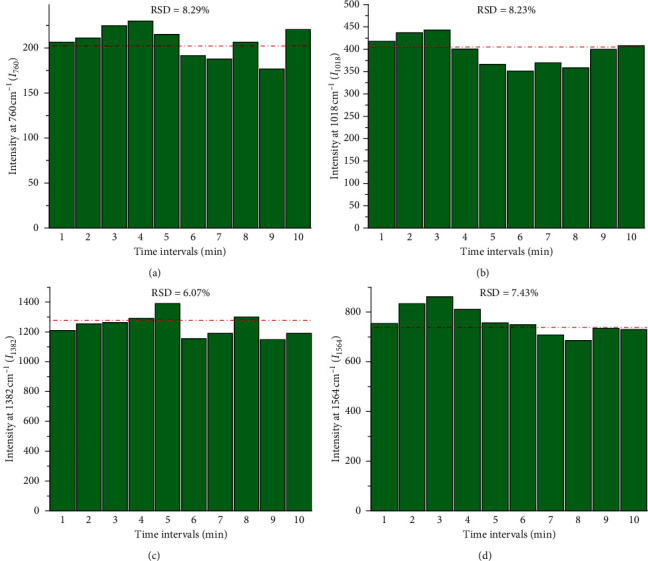
RSD of (a) *I*_760_, (b) *I*_1018_, (c) *I*_1382_, and (d) *I*_1564_ of NAP randomly collected at time intervals of 1 min.

The random aggregation of metal nanoparticles restricts their application for SERS. The sp2 structure and the function of “thermal shielding” from GO make the target compound homogeneously adsorbed on the surface of GO and significantly prevent aggregation, thus improving the stability of metal and graphene composites [37, 38]. In this study, the time-dependent stability of the composite substrates was estimated by RSD of the characteristic peak intensity of NAP (*I*_760_, *I*_1018_, *I*_1382_, and *I*_1564_) at the concentration of 100 ng mL^−1^ randomly collected at various time intervals of 10 min. The RSD is varied between 6.07 and 8.29% (Figure 6), demonstrating that the substrates can produce stable SERS signals with at least 10 min.

**Figure 7:**
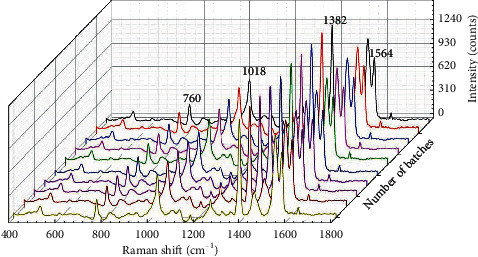
The SERS spectra of NAP measured from 10 different batches of prepared rGO/AuNP. The characteristic peaks of NAP (Δ*v* = 760, 1018, 1362, and 1564 cm^−1^) were marked with red font.

To investigate the reproducibility of the rGO/AuNP substrates, ten different batches of substrates were prepared simultaneously in the same manner and then the SERS performance of the substrates between different batches was measured (Figure 7). The RSD of four characteristic peak intensities of NAP (*I*_760_, *I*_1018_, *I*_1382_, and *I*_1564_) varied from 12.28% to 14.03%.

### 3.4. Real Sample Detection

**Figure 8:**
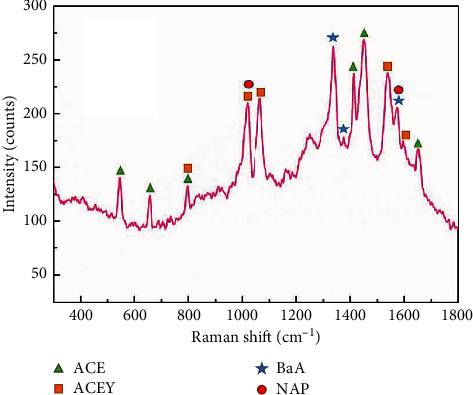
The SERS spectrum of the extract solution from the fried food sample (youtiao). The symbols indicated on the peaks correspond to the respective PAHs.

The SERS-based method has focused on the detection of PAHs in river water [39], soil [22], or as purity compounds [40]. Very few studies have investigated their presence in fried foods. We made an attempt to investigate the analytical potential of the facile SERS substrates developed in this study, for the detection of PAHs in youtiao, a Chinese traditional fried bread commonly consumed for breakfast. A youtiao sample (no. 1349A1-1) was purchased from the local food stall and the pretreatment was performed as described in the section on “Fried Food Sample Detection.” Figure 8 shows that the key SERS peaks of four PAHs (ACE, ACEY, BaA, and NAP) can be easily distinguished with high resolution, without any background interference. The whole detection process takes only about 15 min. The results demonstrate the high potential of the on-field multicomponent detection of PAHs.

## 4. Conclusion

In the present study, a novel SERS sensor was developed for the detection of 16 EPA priority PAHs. The rGO/AuNP substrate can be synthesized without any complicated modification process. The hydrophobic PAHs could be identified based on their characteristic peaks in the presence of the rGO/AuNP composite substrate with estimated LOD as low as 0.2–2 ng·mL^−1^. The different models including linear regression, binary linear regression, and the internal reference methods were optimized for the quantitative calculation of PAHs. The prepared rGO/AuNP sensor platform was preliminarily investigated for the identification of PAHs in Chinese traditional fried food (youtiao) matrix without any complicated pretreatment. The developed SERS-based sensor could prospectively be applied as a screening monitoring method to detect PAHs on-site for the quality control of fried food.

## Figures and Tables

**Table 1:** The 16 EPA priority PAHs.

Chemical name	Abbreviation	CAS no.	Molecular structure
Naphthalene	NAP	91-20-3	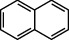

Pyrene	PYR	129-00-0	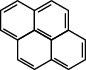

Acenaphthene	ACE	83-32-9	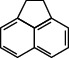

Acenaphthylene	ACEY	208-96-8	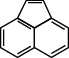

Anthracene	ANTH	120-12-7	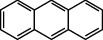

Chrysene	CHR	218-01-9	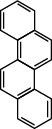

Fluorene	FLU	86-73-7	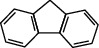

Phenanthrene	PHE	85-01-8	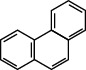

Benzo(a)pyrene	BaP	50-32-8	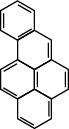

Fluoranthene	FLUA	206-44-0	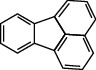

Benz(a)anthracene	BaA	56-55-3	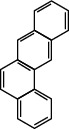

Dibenz(a, h)anthracene	DiB	53-70-3	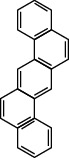

Benzo(b)fluoranthene	BbF	205-99-2	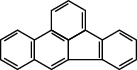

Benzo(k)fluoranthene	BkF	207-08-9	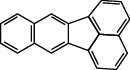

Benzo(g, h, i)perylene	BghiP	191-24-2	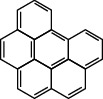

Indeno(1, 2, 3-cd)pyrene	Ind	193-39-5	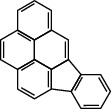

## Data Availability

The data used to support the findings of this study are included within the article.

## Supplementary Materials

Supplementary Materials Figure S1: SERS spectra of 16 PAHs (*C* = 100 ng·mL^−1^) in the presence of rGO/AuNP (red lines) and AuNPs (green lines) substrates. Table S1: band assignments for experimental SERS of NAP. Table S2: band assignments for experimental SERS of PYR. Table S3: band assignments for experimental SERS of ACE. Table S4: band assignments for experimental SERS of ACEY. Table S5: band assignments for experimental SERS of ANTH. Table S6: band assignments for experimental SERS of CHR. Table S7: band assignments for experimental SERS of FLU. Table S8: band assignments for experimental SERS of PHE. Table S9: band assignments for experimental SERS of BaP. Table S10: band assignments for experimental SERS of FLUA. Table S11: band assignments for experimental SERS of BaA. Table S12: band assignments for experimental SERS of DiB. Table S13: band assignments for experimental SERS of BbF. Table S14: band assignments for experimental SERS of BkF. Table S15: band assignments for experimental SERS of BghiP. Table S16: band assignments for experimental SERS of Ind. Table S17: the LOD and quantitative calculation model of 16 PAHs.
