# Supplementary material for: A Versatile SERS Sensor for Multiple Determinations of Polycyclic Aromatic Hydrocarbons and Its Application Potential in Analysis of Fried Foods
Source: Int J Anal Chem. 2020 Aug 3;2020:4248029. doi: 10.1155/2020/4248029 (PMC7397739; doi:10.1155/2020/4248029)
Supplement: Supplementary Materials — Figure S1: SERS spectra of 16 PAHs (C = 100 ng·mL−1) in the presence of rGO/AuNP (red lines) and AuNPs (green lines) substrates. Table S1: band assignments for experimental SERS of NAP. Table S2: band assignments for experimental SERS of PYR. Table S3: band assignments for experimental SERS of ACE. Table S4: band assignments for experimental SERS of ACEY. Table S5: band assignments for experimental SERS of ANTH. Table S6: band assignments for experimental SERS of CHR. Table S7: band assignments for experimental SERS of FLU. Table S8: band assignments for experimental SERS of PHE. Table S9: band assignments for experimental SERS of BaP. Table S10: band assignments for experimental SERS of FLUA. Table S11: band assignments for experimental SERS of BaA. Table S12: band assignments for experimental SERS of DiB. Table S13: band assignments for experimental SERS of BbF. Table S14: band assignments for experimental SERS of BkF. Table S15: band assignments for experimental SERS of BghiP. Table S16: band assignments for experimental SERS of Ind. Table S17: the LOD and quantitative calculation model of 16 PAHs. [file 4248029.f1.docx]

**Supplementary material**

**A versatile SERS sensor for multiple determination of Polycyclic Aromatic Hydrocarbons and its application potential in analysis of fried foods**

Shi Wang ^1^, Jie Cheng ^1,^ *, Caiqin Han ^2^, and Jianchun Xie ^3^

^1^ Institute of Quality Standards and Testing Technologies for Agro-products, Chinese Academy of Agricultural Sciences, Beijing, 100081, China

^2^ Jiangsu Key Laboratory of Advanced Laser Materials and Devices, School of Physics and Electronic Engineering, Jiangsu Normal University, Xuzhou, 221116, China

^3^ Beijing Advanced Innovation Center for Food Nutrition and Human Health, Beijing Technology & Business University (BTBU), Beijing 100048, Beijing

Correspondence should be addressed to Jie Cheng; chengjie@caas.cn;

ORCID: 0000-0003-0773-0244

**Table of contents**

**Fig. S1** SERS spectra of 16 PAHs (*C* = 100 ng·mL^-1^) in the presence of ro-GO/AuNP (red lines) and AuNPs (green lines) substrates.

**Table S1**. Band assignments for experimental SERS of NAP

**Table S2**. Band assignments for experimental SERS of PYR

**Table S3**. Band assignments for experimental SERS of ACE

**Table S4**. Band assignments for experimental SERS of ACEY

**Table S5**. Band assignments for experimental SERS of ANTH

**Table S6**. Band assignments for experimental SERS of CHR

**Table S7**. Band assignments for experimental SERS of FLU

**Table S8**. Band assignments for experimental SERS of PHE

**Table S9**. Band assignments for experimental SERS of BaP

**Table S10**. Band assignments for experimental SERS of FLUA

**Table S11**. Band assignments for experimental SERS of BaA

**Table S12**. Band assignments for experimental SERS of DiB

**Table S13**. Band assignments for experimental SERS of BbF

**Table S14**. Band assignments for experimental SERS of BkF

**Table S15**. Band assignments for experimental SERS of BghiP

**Table S16**. Band assignments for experimental SERS of Ind

**Table S17.** The LOD and quantitative calculation model of 16 PAHs

**References**

**
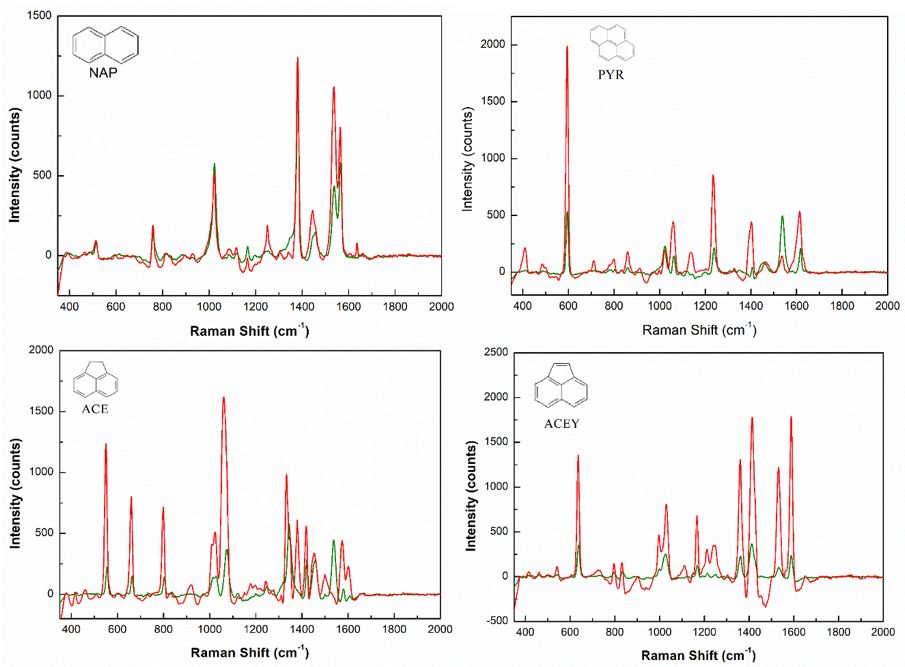
**


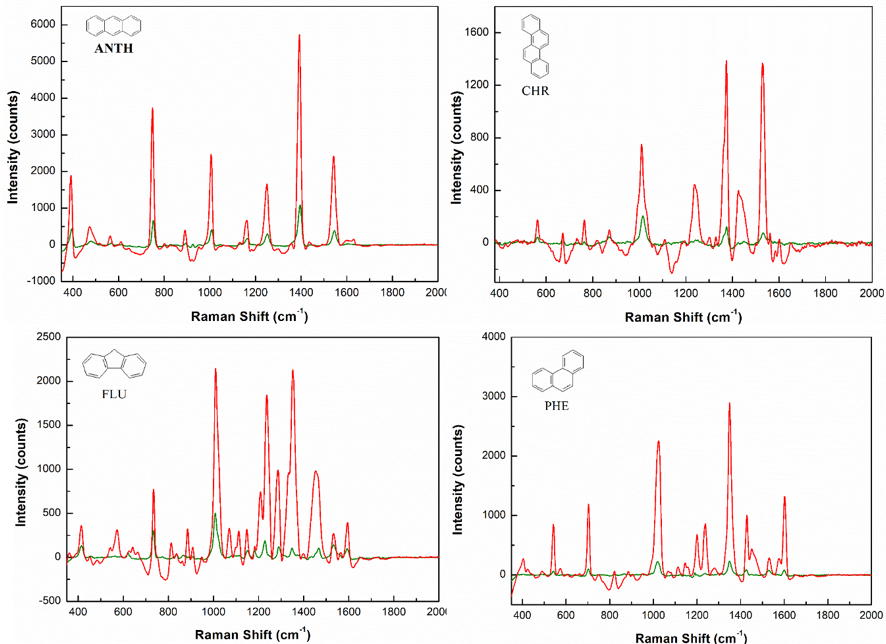


**
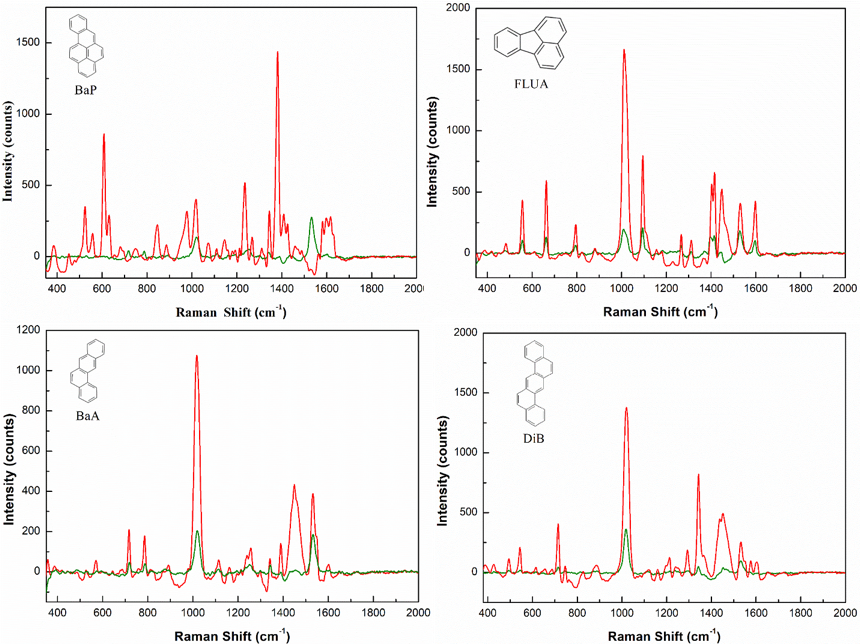
**

**
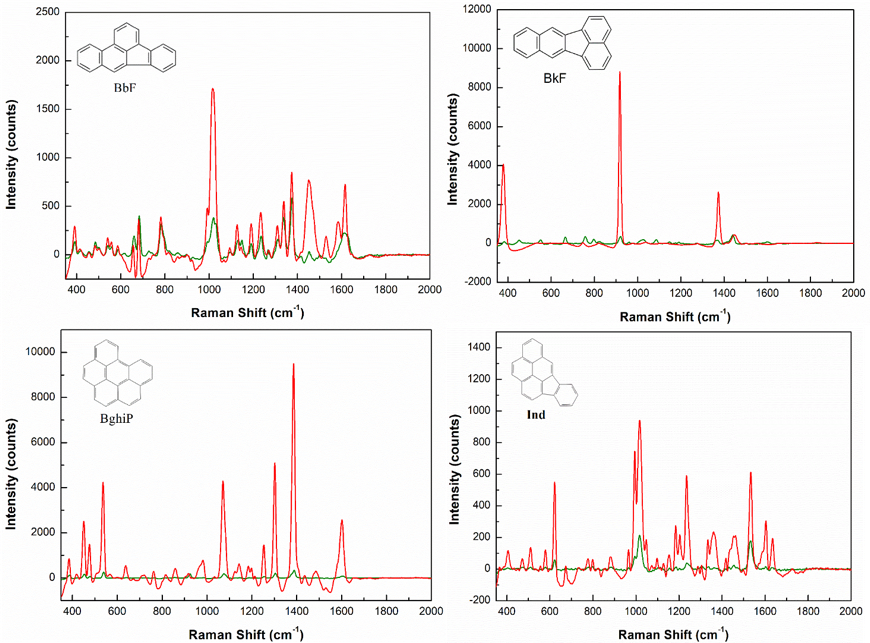
**

**Fig. S1** SERS spectra of 16 PAHs (*C* = 100 ng·mL^-1^) in the presence of ro-GO/AuNP (red lines) and AuNPs (green lines) substrates.

**Table S1**. Band assignments for experimental SERS of NAP

| Experimental  SERS | SERS Ref.^1^ | Vibrational modes |
| --- | --- | --- |
| 512 | 502 | α_C-C-C_ |
| 760 | 754 | γ_C-H_ |
| 1018 | 1015 | ν_C-C_ |
| 1165 | - | β_C-H_*τ*_C-C-C-H_ |
| 1382 | 1378 | ν_C-C_ |
| 1564 | 1561 | ν_C-C_ |

**Table S2**. Band assignments for experimental SERS of PYR

| Experimental  SERS | SERS Ref. ^1^ | Vibrational modes |
| --- | --- | --- |
| 409 | 405 | ν_C-C-C-C_ |
|  | 453 | ν_C-C-C-C_ |
| 486 | 495 | ν_C-C-C-C_ |
| 594 | 592 | ν_C-C-C-C_ |
| 715 |  |  |
| 799 |  |  |
| 859 |  |  |
| 1059 | 1055 | δ_C-H_ |
| 1138 | 1135 | δ_C-H_ |
| 1234 | 1241 | ν_C-C_δ_C-H_ |
| 1400 | 1398 | ν_C-C_*τ*_C-C-C-H_ |
| 1535 |  |  |
| 1612 | 1610 | ν_C-C_ |

**Table S3**. Band assignments for experimental SERS of ACE

| Experimental  SERS | SERS Ref. ^1^ | Vibrational modes |
| --- | --- | --- |
| 416 | 415 | ν_C-C-C-C_ |
| 461 | - | δ_C-H_ |
| 510 | - | ν_C-C-C-C_ |
| 550 | 543 | ν_C-C-C-C_ |
|  | 639 | τ_C-C-C-H_ |
| 660 |  |  |
| 798 | 800 | ν_C-C-C-C_ |
| 836 | 833 | δ_C-H_ |
|  | 1002 | ν_C-C_δ_C-H_ |
|  | 1035 | ν_C-C_δ_C-H_ |
| 1177 | - | δ_C-H_ |
| 1198 | 1215 | ν_C-C_δ_C-H_ |
| 1243 | 1265 ^2^ | ν_C-C_δ_C-H_ |
|  | 1366 | ν_C-C_δ_C-H_ |
| 1418 | 1420 | ν_C-C_δ_C-H_ |
| 1454 | 1461 ^2^ | ν_C-C_δ_C-H_ |
| 1600 | 1593 | ν_C-C_δ_C-H_ |

**Table S4**. Band assignments for experimental SERS of ACEY

| Experimental  SERS | SERS Ref. ^1^ | Vibrational modes |
| --- | --- | --- |
|  | 377 | δ_C-H_ |
| 412 | 414 | ν_C-C-C-C_ |
| 537 | 549 | ν_C-C-C-C_ |
| 634 | 659 | τ_C-C-C-H_ |
| 796 | 800 | ν_C-C-C-C_ν_C-C_ |
| 832 |  |  |
|  | 921 | δ_C-H_ |
| 1000 | 1011 | ν_C-C_δ_C-H_ |
| 1030 | 1026 | ν_C-C_δ_C-H_ |
|  | 1074 | ν_C-C_δ_C-H_ |
| 1165 |  |  |
| 1213 | 1207 | ν_C-C_δ_C-H_ |
| 1247 | 1245 | ν_C-C_δ_C-H_ |
| 1366 | 1350 | ν_C-C_δ_C-H_ |
|  | 1380 | ν_C-C_δ_C-H_ |
| 1409 | 1420 | ν_C-C_δ_C-H_ |
|  | 1460 | ν_C-C_δ_C-H_ |
| 1531 | 1539 | ν_C-C_δ_C-H_ |
| 1591 | 1602 | ν_C-C_δ_C-H_ |

**Table S5**. Band assignments for experimental SERS of ANTH

| Experimental  SERS | SERS Ref. ^1^ | Vibrational modes |
| --- | --- | --- |
| 393 | 392 | ν_C-C-C-C_ |
| 749 | 753 | ν_C-C_ |
| 893 | 891 ^2^ | *τ*_C-C-C-H_ |
| 1008 | 1008 | ν_C-C_ |
| 1252 | 1252 | *τ*_C-C-C-H_ |
| 1393 | 1395 | ν_C-C_*τ*_C-C-C-H_ |
| 1541 | 1545 | ν_C-C_ |

**Table S6**. Band assignments for experimental SERS of CHR

| Experimental  SERS | SERS Ref. ^1^ | Vibrational modes |
| --- | --- | --- |
| 378 | 381 | δ_C-H_ |
| 562 | 568 | ν_C-C-C-C_ |
| 668 |  |  |
|  | 678 | ν_C-C-C-C_τ_C-C-C-H_ |
| 763 | 768 | ν_C-C-C-C_ |
|  | 877 | ν_C-C-C-C_ν_C-C_ |
| 1012 | 1016 | ν_C-C-C-C_τ_C-C-C-H_ |
|  | 1040 | ν_C-C_δ_C-H_ |
| 1238 | 1245 | ν_C-C_δ_C-H_ |
|  | 1334 | ν_C-C_δ_C-H_ |
| 1376 | 1377 | ν_C-C_δ_C-H_ |
| 1427 | 1428 | ν_C-C_δ_C-H_ |
| 1531 |  |  |
|  | 1568 | ν_C-C_δ_C-H_ |
|  | 1605 | ν_C-C_δ_C-H_ |

**Table S7**. Band assignments for experimental SERS of FLU

| Experimental  SERS | SERS Ref. ^1^ | Vibrational modes |
| --- | --- | --- |
| 415 | 416 | ν_C-C-C-C_ |
| 735 | 734 | ν_C-C-C-C_ν_C-C_ |
| 811 | 806 ^2^ | C-H out-of plane bending, C-H wagging |
| 887 | 890 ^2^ | C-H out-of-plane bending, C-H twisting |
| 1009 | 1008 | ν_C-C_δ_C-H_ |
| 1070 | 1054 ^2^ | ν_C-C_δ_C-H_ |
| 1107 | 1108 ^2^ | -- |
| 1150 | 1153 | δ_C-H_ |
| 1207 | 1210 ^2^ | ν_C-C_δ_C-H_ |
| 1238 | 1236 | ν_C-C_δ_C-H_ |
| 1287 | 1299 | ν_C-C_δ_C-H_ |
| 1354 | 1354 | ν_C-C_δ_C-H_ |
| 1455 | 1469 | ν_C-C_δ_C-H_ |
| 1531 | 1545 ^2^ | -- |
|  | 1568 | ν_C-C_δ_C-H_ |
| 1595 | 1601 | ν_C-C_δ_C-H_ |

**Table S8**. Band assignments for experimental SERS of PHE

| Experimental  SERS | SERS Ref. ^1^ | Vibrational modes |
| --- | --- | --- |
| 402 | 405 | ν_C-C-C-C_ |
| 540 | 545 | ν_C-C-C-C_ |
| 704 | 711 | ν_C-C-C-C_ |
| 824 | 826 | ν_C-C-C-C_ |
| 1021 |  |  |
|  | 1032 | ν_C-C_δ_C-H_ |
|  | 1165 | ν_C-C_δ_C-H_ |
| 1200 | 1207 | τ_C-C-C-H_δ_C-H_ |
| 1237 | 1244 | ν_C-C_δ_C-H_ |
| 1351 | 1352 | ν_C-C_δ_C-H_ |
| 1428 | 1431 | ν_C-C_δ_C-H_ |
| 1533 |  |  |
| 1606 | 1610 | ν_C-C_δ_C-H_ |

**Table S9**. Band assignments for experimental SERS of BaP

| Experimental  SERS | SERS Ref. ^1^ | Vibrational modes |
| --- | --- | --- |
|  | 334 | δ_C-C_δ_C-H_ |
| 385 | 381 | δ_C-C_δ_C-H_ |
| 455 | 452 | *τ*_C-C_γ_C-H_ |
| 528 |  |  |
| 555 | 558 | δ_C-C_ δ_C-H_ |
| 608 | 613 | δ_C-C_δ_C-H_ |
| 632 | 636 | *τ*_C-C_δ_C-C_δ_C-H_ |
| 842 | 847 | ν_C-C_ν_C-H_ |
| 979 | 985 | δ_C-H_ν_C-C_ |
| 1016 | 1016 | δ_C-H_ν_C-C_ |
|  | 1196 | δ_C-H_ |
|  | 1216 | δ_C-H_ |
| 1238 | 1238 | δ_C-H_*τ*_C-C-C-H_ |
| 1268 | 1270 | δ_C-H_ |
|  | 1321 | δ_C-H_*v*_C-C_ν_C-C-C-C_ |
| 1348 | 1350 | δ_C-H_*v*_C-C_*τ*_C-C-C-C_ |
| 1378 | 1382 | *v*_C-C-C_ |
| 1406 | 1407 | δ_C-H_*v*_C-C_ |
| 1424 | 1425 | δ_C-H_*v*_C-C_ |
|  | 1572 | δ_C-H_*v*_C-C_ |
|  | 1617 | δ_C-H_*v*_C-C_ |
|  | - | δ_C-H_*v*_C-C_ |

**Table S10**. Band assignments for experimental SERS of FLUA

| Experimental  SERS | SERS Ref. ^1^ | Vibrational modes |
| --- | --- | --- |
|  | 352 | ν_C-C-C-C_ |
|  | 451 | ν_C-C-C-C_ |
| 482 | 484 | ν_C-C-C-C_ν_C-C_ |
| 556 | 563 | ν_C-C-C-C_ |
| 662 | 672 | ν_C-C-C-C_ν_C-C_ |
| 796 | 802 | ν_C-C-C-C_ν_C-C_ |
| 1006 | 1012 | ν_C-C_δ_C-H_ |
| 1098 | 1100 | ν_C-C_δ_C-H_ |
|  | 1158 | ν_C-C_δ_C-H_ |
| 1268 | 1280 | ν_C-C_δ_C-H_ |
| 1311 |  |  |
|  | - | τ_C-C-C-H_ν_C-C_δ_C-H_ |
|  | 1419 | ν_C-C_δ_C-H_ |
| 1448 | 1448 | ν_C-C_δ_C-H_ |
| 1537 | 1544 | ν_C-C_δ_C-H_ |
| 1601 | 1601 | ν_C-C_δ_C-H_ |

**Table S11**. Band assignments for experimental SERS of BaA

| Experimental  SERS | SERS Ref. ^1^ | Vibrational modes |
| --- | --- | --- |
|  | 306 | δ_C-C_ |
| 360 | 358 | δ_C-C_ |
| 528 |  |  |
| 568 | 574 | τ_C-C_ |
| 717 | 722 | ν_C-C_δ_C-C_ |
| 784 | 792 | - |
| 887 |  |  |
| 1019 | 1012 | τ_C-H_ |
|  | 1035 | δ_C-H_ |
| 1116 |  |  |
| 1162 | 1166 | δ_C-H_ |
| 1259 | 1262 | δ_C-H_ |
| 1344 | 1345 | δ_C-C_δ_C-H_ |
| 1388 | 1393 | δ_C-H_ |
| 1448 | 1432 | δ_C-C_δ_C-H_ |
| 1531 |  |  |
|  | 1554 | ν_C-C_δ_C-H_ |
| 1598 | 1610 | ν_C-C_δ_C-H_ |

**Table S12**. Band assignments for experimental SERS of DiB

| Experimental  SERS | SERS Ref. ^1^ | Vibrational modes |
| --- | --- | --- |
|  | 330 | δ_C-H_ |
| 495 | 496 | ν_C-C-C-C_ |
| 544 | 547 | δ_C-H_ |
| 616 | 619 | ν_C-C-C-C_ |
| 714 |  |  |
| 745 | 752 | δ_C-H_ |
|  | 931 | ν_C-C-C-C_δ_C-H_ |
| 1015 |  |  |
|  | 1035 | ν_C-C-C-C_δ_C-H_ |
| 1159 | 1164 | ν_C-C_δ_C-H_ |
| 1214 | 1217 | ν_C-C_δ_C-H_ |
|  | 1260 | ν_C-C_δ_C-H_ |
| 1293 | 1298 | ν_C-C_δ_C-H_ |
| 1342 | 1346 | ν_C-C_δ_C-H_ |
|  | 1380 | ν_C-C_δ_C-H_ |
| 1436 | 1435 | δ_C-H_ |
|  | 1496 | ν_C-C_δ_C-H_ |
| 1531 |  |  |
|  | 1559 | ν_C-C_ |
| 1574 | 1582 | ν_C-C_δ_C-H_ |
| 1604 | 1608 | ν_C-C_δ_C-H_ |

**Table S13**. Band assignments for experimental SERS of BbF

| Experimental  SERS | SERS Ref. ^1^ | Vibrational modes |
| --- | --- | --- |
|  | 334 | ν_C-C-C-C_ |
| 390 | 392 | ν_C-C-C-C_ |
|  | 486 | ν_C-C-C-C_ |
|  | 592 | ν_C-C-C-C_ |
|  | 660 | ν_C-C-C-C_ |
| 684 | 688 | ν_C-C-C-C_ |
| 778 | 790 | ν_C-C-C-C_ |
|  | 901 | δ_C-H_ |
|  |  |  |
| 1015 | 996 | τ_C-C-C-H_ |
|  | 1032 | ν_C-C_ |
|  | 1096 | ν_C-C_δ_C-H_ |
| 1128 |  |  |
| 1192 | 1194 | ν_C-C_δ_C-H_ |
| 1235 | 1238 | τ_C-C-C-H_δ_C-H_ |
|  | 1276 | ν_C-C_δ_C-H_ |
|  | 1314 | ν_C-C_δ_C-H_ |
|  | 1337 | ν_C-C_δ_C-H_ |
| 1376 | 1380 | ν_C-C_δ_C-H_ |
|  | 1418 | ν_C-C_δ_C-H_ |
| 1455 | 1455 | ν_C-C_δ_C-H_ |
| 1533 | 1526 | τ_C-C-C-H_δ_C-H_ν_C-C_ |
|  | 1593 | ν_C-C_δ_C-H_ |
| 1613 | 1620 | ν_C-C_δ_C-H_ |

**Table S14**. Band assignments for experimental SERS of BkF

| Experimental  SERS | SERS Ref. ^1^ | Vibrational modes |
| --- | --- | --- |
|  | 452 | ν_C-C-C-C_τ_C-C-C-H_ |
|  | 492 | ν_C-C-C-C_ |
|  | 552 | ν_C-C-C-C_ |
|  | 601 | δ_C-H_ |
|  | 669 | ν_C-C-C-C_ |
|  | 761 | δ_C-H_ |
|  | 800 | ν_C-C-C-C_ |
|  | 817 | ν_C-C-C-C_δ_C-H_ |
|  | 895 | τ_C-C-C-H_δ_C-H_ |
| 920 | 924 | δ_C-H_ |
|  | 1024 | ν_C-C_δ_C-H_ |
|  | 1031 | ν_C-C_δ_C-H_ |
|  | 1092 | ν_C-C_δ_C-H_ |
|  | 1132 | ν_C-C_δ_C-H_ |
|  | 1153 | ν_C-C_δ_C-H_ |
|  | 1194 | ν_C-C_δ_C-H_ |
|  | 1275 | ν_C-C_δ_C-H_ |
|  | 1342 | ν_C-C_δ_C-H_ |
| 1375 | 1366 | ν_C-C_δ_C-H_ |
|  | 1401 | ν_C-C_δ_C-H_ |
| 1441 | 1442 | ν_C-C_δ_C-H_ |
|  | 1495 | ν_C-C_δ_C-H_ |

**Table S15**. Band assignments for experimental SERS of BghiP

| Experimental  SERS | SERS Ref. ^1^ | Vibrational modes |
| --- | --- | --- |
|  | 366 | ν_C-C-C-C_ |
| 382 | 387 | ν_C-C-C-C_ |
| 420 | 416 | ν_C-C_δ_C-H_ |
| 452 | 452 | *τ*_C-C-C-H_ |
| 478 | 479 | ν_C-C-C-C_ |
| 539 | 538 | *τ*_C-C-C-H_ |
| 637 |  |  |
|  | 712 | ν_C-C_δ_C-H_ |
| 761 |  |  |
| 860 | 848 | ν_C-C_δ_C-H_ |
|  |  |  |
| 979 | 985 | *τ*_C-C-C-H_ |
| 1071 | 1083 | *τ*_C-C-C-H_ |
| 1141 | 1146 | δ_C-H_ |
|  | 1202 | *τ*_C-C-C-H_δ_C-H_ |
| 1255 | 1255 | *τ*_C-C-C-H_δ_C-H_ |
| 1305 | 1305 | ν_C-C_δ_C-H_ |
| 1368 |  |  |
|  | 1385 | ν_C-C_δ_C-H_ |
| 1432 | 1436 | *τ*_C-C-C-H_δ_C-H_ |
| 1599 | 1594 | ν_C-C_ |

**Table S16**. Band assignments for experimental SERS of Ind

| Experimental  SERS | SERS Ref. ^1^ | Vibrational modes |
| --- | --- | --- |
|  | 319 | ν_C-C-C-C_ |
|  | 365 | ν_C-C-C-C_ |
| 407 | 407 | ν_C-C-C-C_ |
| 473 | 473 | ν_C-C-C-C_ |
| 514 |  |  |
| 579 |  |  |
| 623 | 626 | ν_C-C-C-C_ |
| 673 | 680 | ν_C-C-C-C_ |
| 780 | 785 | ν_C-C_*τ*_C-C-C-H_ |
| 799 |  |  |
| 883 |  |  |
|  | 971 | ν_C-C_*τ*_C-C-C-H_ |
|  | 1001 | *τ*_C-C-C-H_ |
| 1017 |  |  |
|  | 1052 | ν_C-C_*τ*_C-C-C-H_ |
|  | 1103 | ν_C-C_δ_C-H_ |
|  | 1153 | ν_C-C_δ_C-H_ |
| 1183 | 1187 | δ_C-H_ |
|  | 1209 | ν_C-C_δ_C-H_ |
| 1233 | 1242 | *τ*_C-C-C-H_δ_C-H_ |
|  | 1295 | ν_C-C_δ_C-H_ |
|  | 1311 | ν_C-C_δ_C-H_ |
|  | 1337 | ν_C-C_*τ*_C-C-C-H_ |
|  | 1380 | ν_C-C_δ_C-H_ |
|  | 1428 | ν_C-C_δ_C-H_ |
|  | 1477 | ν_C-C_δ_C-H_ |
| 1533 | 1538 | ν_C-C_ |
| 1599 | 1587 | ν_C-C_ |
|  | 1606 | ν_C-C_ |
| 1637 | 1645 | ν_C-C_ |

**Table S17. The LOD and quantitative calculation model of 16 PAHs**

| Compound | Fitting regression | *R*^2^ | LOD  (ng·mL^-1^) |
| --- | --- | --- | --- |
| NAP | *C* = -0.5767* *I*_1018_+3.1339* *I*_1564_-748.577 | 0.9989 | 0.5 |
| PYR | *C* = 0.06147* *I*_1234_+0.1143* *I*_1400_+0.4411 | 0.9997 | 0.2 |
| ACE | *C* = 0.1334* *I*_550_+0.0824* *I*_660_+0.6486 | 0.9997 | 1.0 |
| ACEY | *C* = 0.8293* *I*_1213_+0.2180* *I*_1409_-323.123 | 0.9987 | 0.8 |
| ANTH | *C* = 0.09212* *I*_1393_+0.4323* *I*_1548_+4.2894 | 0.9994 | 0.5 |
| CHR | *C* = 0.1326* *I*_1012_+1.8781* *I*_1427_-1.7821 | 0.9990 | 0.8 |
| FLU | *C* = 0.3760* *I*_735_+0.2381* *I*_1354_-0.2421 | 0.9995 | 0.2 |
| PHE | *C* = 0.05268* *I*_704_+0.9253* *I*_1351_+0.4854 | 0.9989 | 0.5 |
| BaP | *C* = 0.4817* *I*_1238_+0.4301* *I*_1378_+1.4213 | 0.9990 | 0.5 |
| FLUA | *C* = 0.2643* *I*_796_+0.0983* *I*_1448_+2.3629 | 0.9894 | 1.0 |
| BaA | *C* = 0.5675* *I*_717_+0.2733* *I*_1531_+0.2690 | 0.9995 | 0.8 |
| DiB | *C* = -0.02649* *I*_714_+2.3462* *I*_1342_+13.3556 | 0.9990 | 0.5 |
| BbF | *C* = 0.3292* *I*_684_+0.3645* *I*_1192_+0.2662 | 0.9901 | 1.0 |
| BkF | *C* = 0.3292* *I*_1375_ +0.6803 | 0.9889 | 2.0 |
| BghiP | *C* = 1.0382* *I*_452_+0.4619* *I*_1305_+0.3305 | 0.9994 | 0.2 |
| Ind | *C* = 0.3534* *I*_623_+0.5347* *I*_1533-_0.15375 | 0.9995 | 0.5 |

**References**

1. J. J. Du, J. W. Xu, Z. L. Sun, and C. Y. Jing, “Au nanoparticles grafted on Fe_3_O_4_ as effective SERS substrates for label-free detection of the 16 EPA priority polycyclic aromatic hydrocarbons,” Analytical Chimica Acta, vol. 915, pp. 81-89, 2016.
2. J. Chen, Y. W. Huang, and Y. P. Zhao, “Characterization of polycyclic aromatic hydrocarbons using Raman and surface-enhanced Raman spectroscopy,” Journal of Raman Spectroscopy, vol. 46, pp. 64-69, 2015.
